# Efficient Recovery Strategy of Luteolin from Agricultural Waste Peanut Shells and Activity Evaluation of Its Functional Biomolecules

**DOI:** 10.3390/ijms241512366

**Published:** 2023-08-02

**Authors:** Seunghee Kim, Kang Hyun Lee, Jeongho Lee, Soo Kweon Lee, Youngsang Chun, Ja Hyun Lee, Hah Young Yoo

**Affiliations:** 1 Department of Biotechnology, Sangmyung University, 20, Hongjimun 2-Gil, Jongno-Gu, Seoul 03016, Republic of Korea; kimseunghee02@naver.com (S.K.); oys7158@naver.com (K.H.L.); jeongholee0601@gmail.com (J.L.); 2 Fermentation Team, Lotte R&D Center, 210 Magokjungang-Ro, Gangseo-Gu, Seoul 07594, Republic of Korea; lsk@lotte.net; 3 Department of Advanced Materials Engineering, Shinhan University, Uijeongbu 11644, Republic of Korea; yschun@shinhan.ac.kr; 4 Department of Convergence Bio-Chemical Engineering, Soonchunhyang University, 22, Soonchunhyang-ro, Asan-si 31538, Republic of Korea

**Keywords:** agriculture waste, peanut shells, bioactive compound, luteolin, optimization

## Abstract

Peanut shells (PSs) generated from agricultural waste contain valuable compounds with bioactive properties such as anti-aging, antimicrobial, and antioxidant properties, making it desirable to recycle them as a sustainable resource. The aim of this study is to design an effective luteolin recovery process as the first step of an integrated biorefinery utilizing PSs as raw material. The major extraction variables and their ranges for luteolin recovery from PSs were determined (0–60 °C, 1–5 h, 0–100% MeOH concentration) and a predictive model was derived through a response surface methodology (RSM). Based on the predictive model, the equation determined for the maximal extraction of luteolin at 1 h was as follows: *y* = –1.8475*x* + 159.57, and the significant range of variables was as follows: 33.8 °C ≤ temperature (*x*) ≤ 48.5 °C and 70.0% ≤ MeOH concentration (*y*) ≤ 97.5%, respectively. High antioxidant and elastase inhibitory activities of PS extracts were confirmed, and these results support their potential to be used as functional materials. In addition, 39.2% of the solid residue after extraction was carbohydrate, which has potential as a carbon source for fermentation. This study provides a useful direction on an integrated biorefinery approach for sustainable agricultural waste valorization.

## 1. Introduction

Rapid population growth and industrialization have accelerated growth of the petrochemical industry. The petrochemical industry is a major contributor to carbon dioxide emissions, causing global warming and contributing to issues such as species loss, sea level rise and abnormal climate [1]. The petrochemical industry is known to account for 30% of global industrial energy use and 16% of carbon dioxide (CO_2_) emissions [2]. The Intergovernmental Panel on Climate Change aims to reach zero CO_2_ emissions by 2050 and restrict the global temperature rise below 1.5 °C by 2100. Accordingly, typical biorefinery has been mainly proposed for bioenergy production through sugar conversion processes including biomass pretreatment, saccharification and fermentation. Furthermore, integrated biorefinery combines different conversion technologies to enable the production of valuable products such as bioactive materials, biofuels and biopolymers [3]. As one of the strategies to realize the integrated biorefinery, a bioactive compound recovery process can be performed prior to the sugar conversion process. Also, for more cost-effective process design, the utilization of industrial waste as feedstock can be an economical strategy. López-Linares et al. reported the potential of spent coffee grounds in the integrated biorefinery concept by recovering antioxidants from spent coffee grounds and then converting the extracted spent coffee grounds into fermentable sugar for butanol production [4]. These strategies can limit environmental pollution and realize a sustainable economy through the valorization of industrial waste and the continuous production of valuable products [5].

Agricultural wastes including shells, seeds, leaves and stems are generated in vast quantities worldwide, and most of them are incinerated or landfilled, causing serious air and soil pollution [6]. It is estimated that the incineration of 1000 kg of agricultural waste releases approximately 1.400 kg of CO_2_, 58 kg of carbon monoxide and 4.9 kg of nitrogen oxides into the atmosphere [7]. However, these wastes have potential as a feedstock in biorefinery due to their valuable components such as bioactive compounds, carbohydrates, lignin and lipids [8]. Peanuts are consumed steadily for the effect of reducing the risk of heart disease, respiratory disease and infectious diseases [9], and many shells are inevitably generated in agricultural processing. In 2017, the global peanut production was estimated at approximately 45 million tons, and about 9.4 million tons of peanut shells (PSs) were generated [10]. It has been reported that PSs are generally incinerated or landfilled, with the exception of some used as feed, plastic stuffing and fuel feedstock [11,12]. PSs contain various bioactive compounds such as luteolin, rutin, naringenin, hesperidin and quercetin as well as cellulose, hemicellulose and lignin [13]. Therefore, PSs have potential as a feedstock for the production of valuable bioproducts, and to realize an integrated biorefinery, an efficient bioactive compound extraction process can be proposed primarily.

Luteolin is a flavonoid compound present in a variety of plant species. It has beneficial biological properties such as antioxidant, antibacterial, anti-inflammatory and anti-cancer properties [14,15,16]. In particular, luteolin has promising therapeutic potential for the treatment of central nervous system diseases due to its ability to cross the blood–brain barrier [17]. In this regard, luteolin is attracting attention from various industries including the food and cosmetic industries. Recently, PSs have attracted attention as a natural source of luteolin. According to several studies related to luteolin recovery, PSs have a relatively high luteolin content (2.41 mg/g-biomass) compared to plant materials such as perilla leaves (0.69 mg/g-biomass) and carrot leaves (0.77 mg/g-biomass) [18,19,20]. Therefore, it is desirable to use PSs for luteolin production through a biorefinery approach.

The purpose of this study is to develop an efficient luteolin recovery process using PSs as the first step of an integrated biorefinery that combines conversion technologies to enable the production of various valuable products from PSs. To select the extraction solvent with the highest recovery of luteolin, PSs were macerated in various solvents. Based on the understanding of the effect of the variables (temperature, time and solvent concentration) on luteolin recovery, an optimal equation was proposed to derive the extraction conditions that could achieve the theoretical maximum luteolin yield in a short time. In order to investigate the potential of PS-derived luteolin in various bio-industries, the bioactive properties (antioxidant and anti-aging) of peanut shell extracts (PSEs) were evaluated. The carbohydrate composition and morphological changes of the solid residue remaining after luteolin extraction were investigated for valorization as a carbon source for microbial fermentation. Finally, our luteolin recovery strategy was compared with related studies that recovered luteolin from various biomass.

## 2. Results and Discussion

### 2.1. Selection of Extraction Solvent for Luteolin Recovery from Peanut Shells

**Figure 1:**
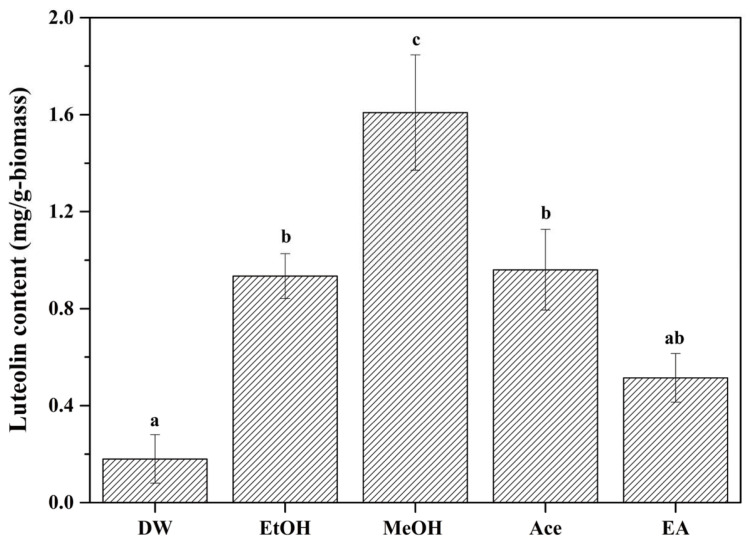
Luteolin content recovered from peanut shells using various solvents. The means within each graph with different letters (a–c) are significantly different (*p* < 0.05).

To select an extraction solvent that enables the efficient recovery of luteolin, peanut shells (PSs) were macerated in various solvents for 2 weeks. Figure 1 shows the luteolin content in the peanut shell extracts (PSEs) after maceration using distilled water (DW), ethanol (EtOH), methanol (MeOH), acetone (Ace), ethyl acetate (EA) and hexane. The results are expressed as luteolin content (mg/g-biomass), defined as the amount of luteolin released from 1 g of PSs. The luteolin contents extracted with DW, EtOH, MeOH, Ace and EA were determined to be 0.18 mg/g-biomass, 0.93 mg/g-biomass, 1.61 mg/g-biomass, 0.96 mg/g-biomass and 0.51 mg/g-biomass, respectively. Hexane was found to be an unsuitable solvent for luteolin extraction because luteolin was not detected in PSE. These results are consistent with Rajhard et al.’s finding that luteolin had higher solubility in MeOH than other solvents [21]. In general, the extraction of phenolic compounds including luteolin is affected by physicochemical properties such as solubility, hydrophobicity and acid–base properties determined by their chemical structure [22,23]. Luteolin has many OH groups in its chemical structure, so it can be a donor or acceptor of hydrogen bonds with other solvents [24]. In this regard, luteolin in PSs would have dissolved by forming hydrogen bonds with polar solvents such as MeOH and EtOH, but not with non-polar solvents such as hexane. Therefore, we finally selected MeOH as an extraction solvent for the efficient recovery of luteolin from PSs.

### 2.2. Optimization of the Extraction Conditions to Derive an Efficient Luteolin Recovery Model

The central composite design (CCD) of response surface methodology (RSM) was utilized to optimize the conditions for luteolin extraction from PSs. PSs were prepared in a size of 90 μm or less using a test sieve. In this study, the CCD was designed by dividing the three variables (*X*_1_; temperature, *X*_2_; time, *X*_3_; MeOH concentration) into five levels (temperature: 0, 15, 30, 45 and 60 °C; time: 1, 2, 3, 4 and 5 h; MeOH concentration: 0, 25, 50, 75 and 100%). The 20 experimental designs and their response values are shown in Table 1. The luteolin yield (%) was selected as the response, and its range was 11.5–92.9%. The luteolin yield was calculated based on the recovered luteolin content (1.61 mg/g-biomass) using 100% MeOH for 2 weeks, which is the theoretical maximal yield (luteolin yield: 100%).

The following model, Equation (1), for the luteolin yield from PSs was predicted through regression analysis on experimental data.

*Y* = 25.58 + 18.00 *X*_1_ + 0.62 *X*_2_ + 18.27 *X*_3_ − 1.30 *X*_1_*X*_2_ + 13.36 *X*_1_*X*_3_ − 0.11 *X*_2_*X*_3_ + 6.74 *X*_1_^2^ + 2.21 *X*_2_^2^ + 5.46 *X*_3_^2^ (1)

where *Y* is the predicted luteolin yield (%) and *X*_1_, *X*_2_ and *X*_3_ are the temperature, time and MeOH concentration, respectively.

Table 2 indicates the analysis of variance (ANOVA) results for the luteolin yield model. ANOVA was used to verify the validity of the experimental results. High F-values and low *p*-values (*p* < 0.05) indicate that the predicted model is mathematically and statistically significant [25]. The F-value and *p*-value of the predicted model were 122.8 and <0.0001, respectively. Also, the model terms *X*_1_ (*p*-value: <0.0001), *X*_3_ (*p*-value: <0.0001), *X*_1_*X*_3_ (*p*-value: <0.0001), *X*_1_^2^ (*p*-value: <0.0001), *X*_2_^2^ (*p*-value: 0.0100) and *X*_3_^2^ (*p*-value: <0.0001) were significant for the luteolin yield. The fit of the model is inspected by the R^2^ and the *p*-value is used for lack of fit [26]. The R^2^ and adjusted R^2^ were determined to be 0.9910 and 0.9830, respectively. The R^2^ and adjusted R^2^ were higher than 0.9 and the difference of the R^2^ and adjusted R^2^ is less than 0.2, meaning that the model sufficiently indicates the experimental results [27]. The coefficient of variation (CV) means the ratio of the standard deviation to the mean, and a low CV value (<10%) means high reproducibility and reliability of the model [28]. The adequate precision (AP) shows the signal-to-noise ratio and represents whether the model can explore the design space [16]. The ratio being higher than 4 indicates the mean of the model is performed appropriately depending on the prediction. The CV and AP of the model were 9.43% and 32.134, respectively, proving that the predicted model has accuracy and reliability. Overall, these ANOVA results demonstrate that the developed model is statistically valid and reliable for predicting luteolin yield.

**Figure 2:**
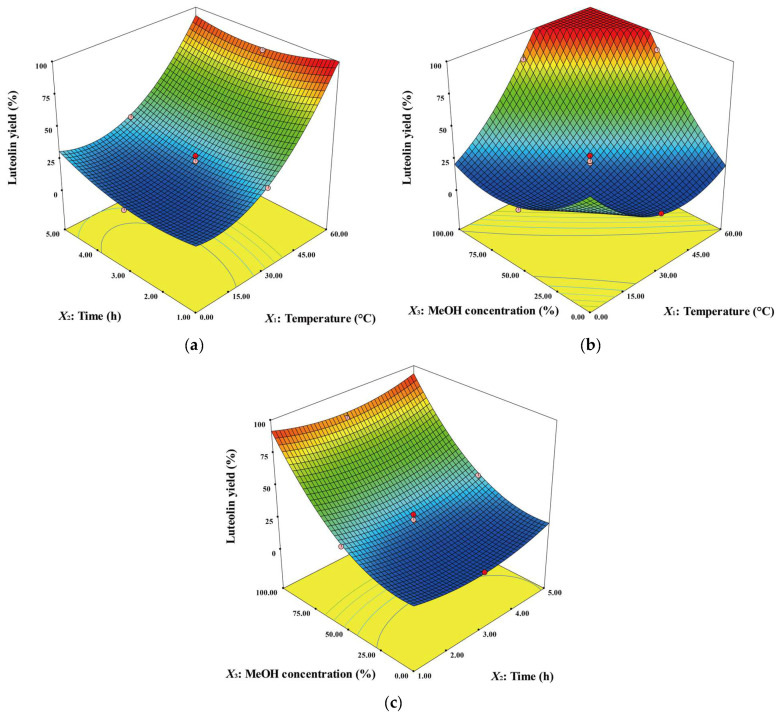
Response surface plots representing the effects of each variable on luteolin yield. The effects of temperature and time (**a**), temperature and MeOH concentration (**b**) and time and MeOH concentration (**c**).

The interaction effects of extraction variables on luteolin yield were investigated, and Figure 2 is a three-dimensional (3D) response surface plot showing their interactions. The plots show the effects of the other two variables on luteolin yield, with one variable fixed at the center point (coded factor level 0). Figure 2a shows the interaction effect of temperature and time on luteolin yield. The effect of time was relatively insignificant in all temperature condition ranges. Conversely, temperature above 30 °C contributed to improved luteolin yield over the entire range of time conditions. Figure 2b shows the interaction effect between temperature and MeOH concentration on luteolin yield. As both the MeOH concentration and temperature increased simultaneously from the center point, the luteolin yield increased rapidly. However, since the maximal yield of the luteolin recovery process cannot exceed 100%, the *y*-axis range of the plot was set to a maximal of 100%. Thus, there are some partially flat plot parts as the levels of the two variables increase significantly. Figure 2c shows the interaction effect between reaction time and MeOH concentration on luteolin yield. Consistent with the results in Figure 2a, reaction time did not significantly affect luteolin recovery. MeOH concentration above 50% contributed to increased luteolin yields over the entire range of reaction time conditions.

Numerical optimization was performed with the goal of maximizing the luteolin yield to derive optimal luteolin recovery conditions. It was carried out by fixing the reaction time to 1 h and setting the reaction temperature to above 30 °C and the MeOH concentration to above 50%. The derived conditions are shown in Table 3. The ranges of temperature and MeOH concentration in the conditions were as follows: 33.8 °C ≤ temperature ≤ 58.6 °C and 51.7% ≤ MeOH concentration ≤ 97.5%.

An inverse correlation was found between temperature and MeOH concentration to achieve the 100% luteolin yield. The inverse relationship between temperature and MeOH concentration is indicated as the linear Equation (2):

*y* = −1.8475*x +* 159.57 (2)

where *y* is MeOH concentration (%) and *x* is temperature (°C). The reliability of Equation (2) was validated through the actual experiments of the selected luteolin extraction conditions (Table 4).

**Figure 3:**
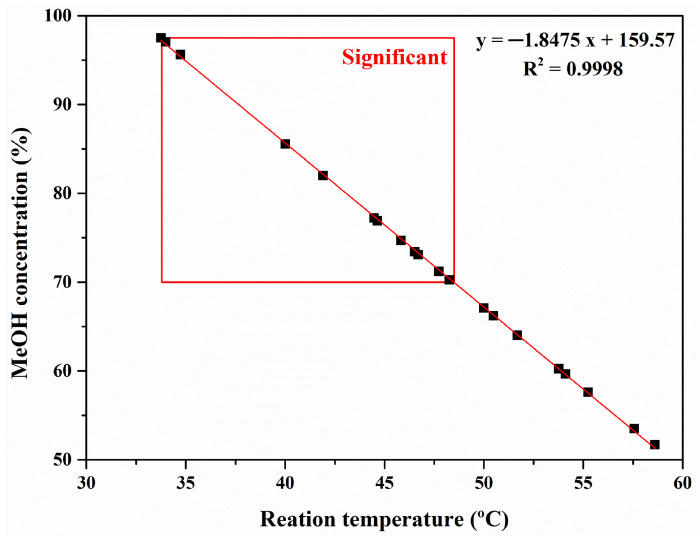
Correlation analysis of temperature and MeOH concentration for the design of a model for maximal luteolin recovery from peanut shells.

Figure 3 shows an advanced significant range of the optimal luteolin extraction conditions precisely predicted using Equation (2) for the maximum luteolin yield. The derived linear equation has advanced significance at temperatures below 48.5 °C and at MeOH concentrations above 70%. In conclusion, the range of advanced extraction conditions to reach the maximal luteolin yield from PSs at 1 h was as follows: 33.8 °C ≤ temperature (*x*) ≤ 48.5 °C and 70.0% ≤ MeOH concentration (*y*) ≤ 97.5%.

### 2.3. Bioactivity Evaluation of Peanut Shell Extracts

To quantify the bioactive compound contents in PSEs recovered under the optimal conditions, total polyphenol, total flavonoid and the luteolin content were analyzed. The total polyphenol and flavonoid contents of PSEs were determined to be 6.6 ± 0.05 mg/g-biomass and 4.1 ± 0.40 mg/g-biomass, respectively (Table 5). The results were consistent with the report of Meng et al., in which luteolin was the main component of the phenolic compounds in PSs [13].

The antioxidant activity of PSEs was analyzed using ferric reducing antioxidant power (FRAP), 2,2′-Azino-bis(3-ethylbenzothiazoline-6-sulfonic acid) (ABTS) and 2,2-diphenyl-1-picrylhydrazyl (DPPH) assays. The FRAP assay measures the transition metal reduction potential and the ABTS and DPPH assays evaluate the radical scavenging activity [29]. The control group was prepared by dissolving a luteolin standard in MeOH at the same concentration as the luteolin concentration in PSEs. Table 6 indicates the antioxidant activity results of the luteolin standard and PSE. The FRAP values of the luteolin standard and PSEs were 3.0 ± 0.08 mmol/L and 4.3 ± 0.03 mmol/L, respectively, indicating that PSEs had about 1.4-fold higher transition metal reducing power than the luteolin standard. The results obtained for the ABTS and DPPH assays are expressed as the half maximal inhibitory concentration (IC_50_). The ABTS IC_50_ and DPPH IC_50_ of the luteolin standard were 17.3 ± 0.08 µg/mL and 174.7 ± 0.34 µg/mL, respectively and the ABTS IC_50_ and DPPH IC_50_ of PSEs were 4.6 ± 0.10 µg/mL, and 11.0 ± 0.44 µg/mL, respectively. In other words, PSEs had 3.8-fold and 15.9-fold higher antioxidant activity than the luteolin standard for ABTS radicals and DPPH radicals, respectively. To evaluate the anti-aging activity of PSEs, the elastase inhibition activity was investigated. The elastase inhibition activity of the luteolin standard and PSEs was 49.9 ± 0.03% and 88.3 ± 0.55%, respectively, indicating that PSEs had about 1.8-fold higher than the luteolin standard. These results are consistent with Geng et al.’s finding that luteolin inhibited the elastase activity of *Pseudomonas aeruginosa* [30]. According to the bioactivity evaluation results, PSEs showed higher antioxidant and elastase inhibitory activity than the luteolin standard. It is believed that PSs contain flavonoids such as rutin, hesperidin and quercetin as well as luteolin [13]. Consequently, the potential of PSs as a bioresource for functional material production was confirmed through various bioactivity evaluations.

### 2.4. Carbohydrate Composition of Peanut Shells and the Residue after Luteolin Extraction

To evaluate the potential of PSs as a feedstock for sugar conversion processes, the changes in the carbohydrate composition and morphology of PS residues after luteolin extraction were investigated. As a result, it was confirmed that PSs and extracted PSs (ePSs) were mainly composed of glucan and xylan. Specifically, PSs were composed of glucan 23.2 ± 0.25%, xylan 9.8 ± 0.03%, arabinan 2.5 ± 0.06% and others 64.5%, and ePSs were composed of glucan 23.6 ± 0.10%, xylan 14.6 ± 0.03%, arabinan 1.0 ± 0.07% and others 60.8% (Table 7). The ratio of carbohydrates was found to increase slightly after the luteolin extraction process, and in particular, xylan was shown to be increased by about 1.5-fold. The increased percentage of carbohydrates is thought to be due to the removal of phenolic compounds (i.e., other substances) during the extraction process.

**Figure 4:**
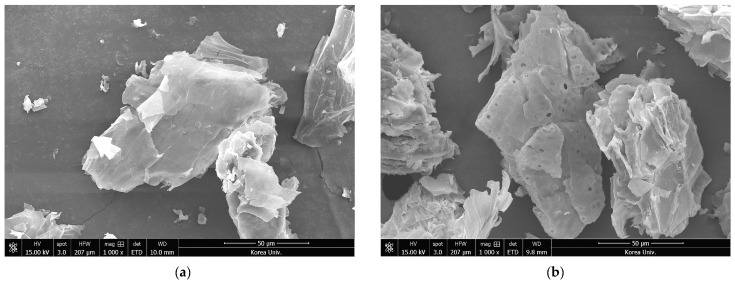
Scanning electron micrographs of untreated peanut shells (**a**) and extracted peanut shells (**b**).

Figure 4 shows the morphological changes of PSs and ePSs. As shown in Figure 4a, the external surfaces of PSs were non-porous and had smooth morphology. Conversely, as shown in Figure 4b, the external surfaces of ePSs were irregular and rough and had porous morphology. According to Ozturk et al., solvent extraction can cause modification of the polysaccharide structure in the cell wall [31] In addition, it has been reported that this modified structure significantly affects sugar conversion by increasing the accessibility of enzymes [32]. These results suggest that the solid residue remaining after luteolin extraction can be utilized as a feedstock.

### 2.5. Evaluation of Overall Process for Luteolin Recovery from Peanut Shells

**Figure 5:**
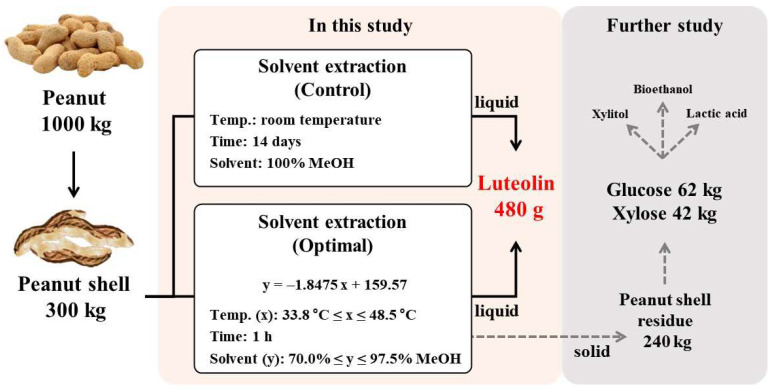
Schematic diagram of the mass balance in the integrated biorefinery process of peanut shells including luteolin recovery.

Figure 5 shows the mass balance of an integrated biorefinery process concept for the recovery of various biochemicals including luteolin from PSs. It is assessed that about 300 kg of PSs can be generated from 1000 kg of peanuts [33]. In the control process (temperature, room temperature; time, 14 days; solvent, 100% MeOH), it is estimated that approximately 480 g of luteolin was recovered based on 300 kg of PSs. In the optimal process derived using the equation *y* = −1.8475 *x* + 159.57 (33.8 °C ≤ temperature (*x*) ≤ 48.5 °C and 70.0% ≤ MeOH concentration (*y*) ≤ 97.5%) with the extraction time at 1 h, it is expected that about 480 g of luteolin can be recovered based on 300 kg of PSs. The objective of both processes is to recover the same amount of luteolin; however, the optimized process significantly reduced the extraction time. Therefore, the proposed optimal process can be used as an economical luteolin recovery strategy. After luteolin extraction, approximately 240 kg of ePS solid residue is generated, which contains carbohydrates that can be converted into fermentable sugars. Based on 240 kg of ePS solid residue, it is estimated that approximately 62 kg of glucose and 42 kg of xylose can be produced. The fermented sugar derived from ePS solid residue can be used as a carbon source in microbial fermentation to produce various high-value-added compounds such as xylitol, bioethanol and lactic acid. In a further study, we plan to develop a process for the recovery of fermentable sugars such as glucose and xylose from ePS solid residue after extraction for the realization of integrated biorefinery.

Recent studies on luteolin recovery from biomass are summarized in Table 8. The luteolin recovery was performed from biomass such as carrot [20], Cretan brake fern [34], deulkkae [19], leaves of olive trees [35,36], pigeon pea [37], PSs [18] and ribwort plantain [38] using various extraction methods. Among them, PSs have a higher luteolin content than any other biomass. Extraction techniques such as enzyme-assisted extraction, hydrothermal extraction, maceration, pressurized liquid extraction, supercritical fluid extraction and ultrasonic-assisted extraction have been utilized to recover luteolin from biomass. The techniques such as ultrasound, supercritical fluid, and pressurized liquid extraction require high temperature, pressure and energy input, increasing the operating cost of the overall process [39]. Maceration is one of the most economical extraction methods used in industry to recover phytochemicals with low extraction efficiency, long extraction times and thermal instability [40]. The luteolin recovery process from PSs proposed in this study has the advantage that it was performed using maceration under mild conditions without additional high energy, high temperature or high pressure.

The novelty of our study is that an optimal predictive model was derived as the maximal luteolin recovery yield from PSs within 1 h. Our predicted model represents a significant range of luteolin recovery conditions by performing validation experiments under predicted conditions that can show theoretical maximal yields. Our strategy for deriving predictive models is believed to be very effective in recovering bioactive substances from biomass. Therefore, this study is expected to contribute to the realization of a sustainable integrated biorefinery using agricultural waste.

## 3. Materials and Methods

### 3.1. Materials

Peanuts were grown on Buyeo 153 farm (Buyeo, Chungcheongnam-do, Korea). The kernels of the peanuts were removed and only the shells were recovered. Peanut shells (PSs) were washed with DW and dried at 110 °C. After that, PSs were prepared with a size of less than 90 μm using a test sieve and used as biomass. Ethanol (EtOH, 94.50%), methanol (MeOH, 99.9%), acetone (Ace, 99.7%) and ethyl acetate (EA, 99.9%) were purchased from Samchun Chemical (Gangnam, Seoul, Republic of Korea). Folin–Ciocalteu reagent, sodium carbonate (Na_2_CO_3_), luteolin, gallic acid, sodium nitrite (NaNO_2_), 2,4,6-tripyridyl-S-triazine (TPTZ), iron (II) sulfate (FeSO_4_), iron (III) chloride (FeCl_3_), sodium acetate trihydrate (CH_3_CO_2_Na·3H_2_O), 1,1-diphenyl-2-picryl-hydrazyl (DPPH), 2,2′-azino-bis (3-ethylbenzothiazoline-6-sulphonic acid) (ABTS), N-Succinyl-Ala-Ala-Ala-p-nitroanilide and elastase from porcine pancreas were obtained from Sigma-Aldrich (St. Louis, MO, USA). Aluminum chloride (AlCl_3_), calcium carbonate (CaCO_3_), hexane (95%) and sulfuric acid (H_2_SO_4_) were purchased from Duksan Pure Chemical (Ansan-si, Gyeonggi-do, Republic of Korea). All reagents used in this study were analytical grade.

### 3.2. Sample Preparation and Solvent Selection

PSs were prepared with a size of 90 μm or less using a test sieve. First, 1 g of PSs was macerated in 10 mL of extract solvent, and the reaction was carried out in a water bath (BHS-2, JOANLAB, Huzhou, China) at 25 °C for 14 days. The extracts were centrifuged at 13,000 rpm for 5 min, and the supernatants were used for luteolin content analysis. All experiments were performed in triplicate.

### 3.3. Experimental Design through Response Surface Methodology

Central composite design (CCD) of response surface methodology (RSM) was carried out to optimize the conditions for luteolin recovery from PSs. CCD provides mathematical models by considering the interaction of variables. The model equation is converted into a quadratic function and the response value is estimated using the variable of the quadratic model. The variables and their ranges were as follows: temperature (*X*_1_; 0–60 °C), time (*X*_2_; 1–5 h) and MeOH concentration (*X*_3_; 0–100%) (Table 9).

Analysis of variance (ANOVA) was carried out to prove the validity of the estimated model. The effects and interactions of variables on the response were determined using the following quadratic Equation (3):

(3) $$Y=\beta_{0}+\sum_{i=1}^{k}\beta_{i}X_{i}+\sum_{i=1}^{k}\beta_{ii}X_{i}^{2}+\sum_{i=1}^{k}\sum_{j=i+1}^{k}\beta_{ij}X_{i}X_{j},\;\;$$

where *Y* is the output response values (luteolin yield), *β*_0_ is the offset term and *β_i_*, *β_ii_* and *β_ij_* are regression model coefficients [41]. *k* indicates the number of variables (*k* = 3 in this study). *X_i_* and *X_j_* denote the input variables values (temperature, time and MeOH concentration). The ANOVA and numerical optimization were carried out using the software Design-Expert version 13 (Stat-Ease, Inc., Minneapolis, MN, USA). All experiments were performed in triplicate, and the results were calculated as an average.

### 3.4. Analytical Procedures

#### 3.4.1. Determination of Total Polyphenol Content

The total polyphenol content of peanut shell extracts (PSEs) was measured using the Folin–Ciocalteu colorimetric method, which was performed following an improved protocol from our previous study [42]. For this, 10 μL of a prepared sample, 790 μL of DW and 50 μL of Folin–Ciocalteu reagent were mixed in a 1.5 mL e-tube using a vortex mixer for about 10 s. The mixture was then allowed to react at 30 °C in a water bath for 8 min. After the reaction, 150 μL of a 20% Na_2_CO_3_ solution was added to the e-tube containing the sample, and the reaction was allowed to proceed for additional 1h at 25 °C in a water bath. Finally, the reaction mixture was transferred into a cuvette and the absorbance was measured at 765 nm using a spectrophotometer (DU^®^ 730, Beckman Coulter, Brea, CA, USA). All experiments were performed in triplicate and the results were expressed as mg gallic acid equivalent (GAE) per g of biomass.

#### 3.4.2. Determination of Total Flavonoid Content

The total flavonoid content of PSEs was measured using a modified aluminum chloride colorimetric method [43]. Briefly, 50 μL of a sample and 30 μL of 5% NaNO_2_ were mixed in an e-tube using a vortex mixer for approximately 10 s and then reacted for 6 min at 25 °C in a water bath. After the reaction, 50 μL of 10% AlCl_3_ solution was added to the e-tube and reacted for 5 min at 25 °C in a water bath. Then, 300 μL of 1 M sodium hydroxide and 1 mL of DW were added to the e-tube, respectively, and mixed using a vortex mixer for about 10 s before reacting for 15 min at 25 °C in a water bath. Finally, the reactant was transferred into a cuvette and the absorbance was determined at 510 nm using the spectrophotometer. All experiments were performed in triplicate, and the total flavonoid content was expressed as mg quercetin equivalent (QE) per gram of biomass.

#### 3.4.3. Determination of Ferric Reducing Antioxidant Power Activity

The antioxidant activity of PSEs was confirmed through modification of the FRAP assay as described by Nkurunziza et al. [44]. To prepare the FRAP working solution, 300 mM sodium acetate buffer (pH 3.6), 20 mM FeCl_3_·6H_2_O and 10 mM TPTZ solution in 40 mM HCl were combined in a volume ratio of 10:1:1. First, a pre-warmed solution of 300 µL DW at 37 °C for 5 min was prepared. Next, 30 µL of PSE and 900 µL of the FRAP working solution were added to the pre-warmed DW and then left at 37 °C for 4 min. Finally, the reactant was transferred into a cuvette and the absorbance was determined at 593 nm, and the blank was made using DW in place of PSE. The standard curve, which allowed for the calculation of FRAP values, was linear between 0.1 and 1 mmol/L ascorbic acid.

#### 3.4.4. Determination of 2,2′-Azino-bis(3-ethylbenzothiazoline-6-sulfonic acid) Free Radical Scavenging Assay

The antioxidant activity of PSEs was confirmed by modifying the ABTS radical scavenging assay [45]. For the preparation of ABTS^•+^ solution, 10 mL of a 7 mM ABTS solution and 10 mL of 2.45 mM K_2_S_2_O_8_ were mixed. Next, 950 μL of the ABTS^•+^ solution was mixed with 50 μL of the sample. The mixture was then vortexed and allowed to react in a water bath at 25 °C for 30 min. Following the reaction, the reactant was transferred into a cuvette and the absorbance was determined at 734 nm using the spectrophotometer. The ABTS^•+^ scavenging activity was quantified using Equation (4) and the results were expressed as IC_50_, which represents the concentration of the extracts required to scavenge 50% of the initial radicals.

ABTS radical scavenging activity (%) = (1 − A_control_/A_sample_) *×* 100 (4)

where A_control_ and A_sample_ represent the absorbance of the control and sample at 734 nm, respectively.

#### 3.4.5. Determination of 2,2-Diphenyl-1-picrylhydrazyl Free Radical Scavenging Assay

The antioxidant activity of PSEs was assessed using a modified DPPH radical scavenging assay, as described by Ndayishimiye et al. [46]. For this, 500 μL of 0.25 mM DPPH was mixed with 500 μL of the sample in an e-tube. The mixture was vortexed and placed in a water bath set at 25 °C for 30 min. Finally, the reactant was transferred into a cuvette and the absorbance was determined at 517 nm using the spectrophotometer. The DPPH free radical scavenging activity was quantified using Equation (5) and the results were expressed as IC_50_.

DPPH radical scavenging activity (%) = (1 − A_control_/A_sample_) *×* 100 (5)

where A_control_ and A_sample_ represent the absorbance of the control and sample at 517 nm, respectively.

#### 3.4.6. Determination of Elastase Inhibition Activity

The elastase inhibition activity of PSEs was measured by modifying the method of Chiocchio et al. [47]. For this, 360 µL of phosphate-buffered saline (PBS) buffer (pH 7.0), 200 µL of PSE and 120 µL of elastase from porcine pancreas were preheated at 37 °C for 10 min. Then, 60 µL of 2 mM N-Succinyl-Ala-Ala-Ala-*p*-nitroanilide was added to the mixture and it was left to react at 37 °C for 20 min. The absorbance of the sample was measured at 410 nm, and the elastase inhibition activity of PSE was calculated using the following Equation (6):

Elastase inhibition activity (%) = [1 − (A_sample_ − A_sample blank_)/A_control_] *×* 100 (6)

where A_sample_, A_sample blank_ and A_control_ represent the absorbance of the control and sample at 410 nm, respectively. The sample blank and control used were PBS.

#### 3.4.7. High-Performance Liquid Chromatography Analysis

The luteolin content of PSEs was quantified using a high-performance liquid chromatography diode array detector (HPLC-DAD, Hitachi, Tokyo, Japan). An INNO Column C18 (5 µm, 4.6 mm × 250 mm, Young Jin Biochrom, Seongnam-si, Korea) was used for the luteolin content analysis at 25 °C. The gradient elution was 100% acetonitrile as mobile phase A and 0.03% phosphoric acid (*v*/*v*) as mobile phase B. The gradient conditions were as follows: start at 10% A and 90% B; 90–80% B, 0–5 min; 80–60% B, 5–20 min; 60–25% B, 20–45 min; 25–90% B, 45–47 min and 90% B, 47–50 min. The flow rate was 0.6 mL/min, the wavelength was 250 nm and the injection volume was 5 µL.

The carbohydrate composition of PSs and extracted PSs (ePSs) was analyzed according to a report described by the National Renewable Energy Laboratory [48]. After extracting luteolin from PSs under optimal conditions, the ePSs were recovered and dried at 110 °C. Next, 0.3 g of PSs, ePSs and each sugar standard (D-(+)glucose, D-(+)xylose, D-(+)galactose, L-(+)arabinose and D-(+)mannose) were saturated in 3 mL of 72% (*w*/*w*) H_2_SO_4_ at 30 °C for 2 h. Then, after diluting with DW to 4% acid concentration, the mixture was reacted at 121 °C for 1 h. Following neutralization with calcium carbonate, the supernatant was filtered through a 0.22 μm syringe filter to prepare a sample for HPLC analysis. The carbohydrate compositions of PSs and extracted PSs (ePSs) were quantified using a Shimadzu HPLC system equipped with a Shimadzu LC-20AT pump, SIL-20A automatic sampler, CTO-20A column oven and RID 10A refractive index detector (Shimadzu, Kyoto, Japan). A Shodex SUGAR SH1011 H^+^ ion exclusion column (8 mm × 300 mm, Shodex, Japan) was used at 50 °C. The mobile phase was 0.005 N H_2_SO_4_, the flow rate was 0.8 mL/min and the injected amount was 20 μL. The carbohydrate composition of the sample was calculated as the ratio between the HPLC analysis results of the sample and the sugar standard.

#### 3.4.8. Morphological Analysis

The surface morphology of PSs and ePSs were analyzed using scanning electron microscopy (SEM, Quanta FEG 250, FEI, Hillsboro, OR, USA). All samples were coated with platinum under vacuum for 120 s using a Cressington Sputter Coater 108 auto (Cressington Scientific Instruments, Watford, UK) before visualization under the SEM. The vacuum value was 8.1 × 10^−7^ mbar. An accelerating voltage of 15 kV was used for imaging and the working distance for PSs and ePSs was 10 mm and 9.8 mm, respectively.

## 4. Conclusions

In this study, we derived an efficient luteolin recovery model from peanut shells (PSs) as a first strategy toward the realization of an integrated biorefinery. The equation leading to the optimal extraction to achieve 100% luteolin yield in a short time (1 h) was as follows: *y* = −1.8475*x* + 159.57, and the ranges of the variables were 33.8 °C ≤ temperature (*x*) ≤ 48.5 °C and 70.0% ≤ MeOH concentration (*y*) ≤ 97.5%, respectively. In future research, we plan to design a second strategy that converts the extracted PSs into fermentable sugar.

## Figures and Tables

**Table 1:** Experimental designs and their response for five-level, three-factor response surface analysis.

Run	Coded Factor Levels			Response
	*X*_1_	*X*_2_	*X*_3_	Luteolin Yield (%)
1	−1	−1	−1	15.0
2	1	−1	−1	28.0
3	−1	1	−1	20.7
4	1	1	−1	26.0
5	−1	−1	1	28.9
6	1	−1	1	92.9
7	−1	1	1	31.7
8	1	1	1	92.8
9	−2	0	0	14.4
10	2	0	0	86.7
11	0	−2	0	31.5
12	0	2	0	33.3
13	0	0	−2	11.5
14	0	0	2	79.3
15	0	0	0	22.1
16	0	0	0	23.6
17	0	0	0	28.1
18	0	0	0	28.1
19	0	0	0	23.8
20	0	0	0	23.8

**Table 2:** Analysis of variance for the developed luteolin recovery model.

Source	Sum of Square	Degree of Freedom	Mean Square	F-Value	*p*-Value	Remarks
Model	13,540.62	9	1504.51	122.8	<0.0001	Significant
*X*_1_	5183.34	1	5183.34	423.0	<0.0001	Significant
*X*_2_	6.24	1	6.24	0.5	0.4917	
*X*_3_	5342.13	1	5342.13	436.0	<0.0001	Significant
*X*_1_ *X*_2_	13.60	1	13.60	1.1	0.3169	
*X*_1_ *X*_3_	1426.99	1	1426.99	116.5	<0.0001	Significant
*X*_2_ *X*_3_	0.097	1	0.097	0.0	0.9309	
*X*_1_^2^	1143.45	1	1143.45	93.3	<0.0001	Significant
*X*_2_^2^	123.06	1	123.06	10.0	0.0100	Significant
*X*_3_^2^	749.39	1	749.39	61.2	<0.0001	Significant
Residual	122.53	10	12.25			
Lack of fit	89.89	5	17.98	2.8	0.1453	Not significant
Pure error	32.65	5	6.53			
Total	13,663.15	19				

Coefficient of determination (R^2^): 0.9910. Adjusted R^2^: 0.9830. Coefficient of variation (CV): 9.43%. Adequate precision (AP): 32. 134.

**Table 3:** Solutions for optimization with luteolin yield of 100%.

	*X*_1_: Temperature (°C)	*X*_3_: MeOH Concentration (%)	Predicted Luteolin Yield (%)	Desirability
1	33.8	97.5	100	1.0
2	34.0	97.1	100	1.0
3	34.7	95.6	100	1.0
4	40.0	85.6	100	1.0
5	41.9	82.0	100	1.0
6	44.5	77.2	100	1.0
7	44.6	76.9	100	1.0
8	45.8	74.7	100	1.0
9	46.5	73.4	100	1.0
10	46.7	73.1	100	1.0
11	47.7	71.2	100	1.0
12	48.3	70.3	100	1.0
13	50.0	67.1	100	1.0
14	50.5	66.2	100	1.0
15	51.7	64.0	100	1.0
16	53.8	60.3	100	1.0
17	54.1	59.7	100	1.0
18	55.3	57.6	100	1.0
19	57.6	53.5	100	1.0
20	58.6	51.7	100	1.0

**Table 4:** Predicted and experimental responses for validation of predictive equation for luteolin recovery within the designed range.

Run	Variables			Response		Remarks
	Temp. (°C)	Time (h)	MeOH Conc. (%)	Luteolin Yield (%)		
				Predicted	Experimental	
1	37.7	1	90	100	97	Significant
2	40.4	1	85	100	96	Significant
3	43.1	1	80	100	99	Significant
4	45.8	1	75	100	98	Significant
5	48.5	1	70	100	96	Significant
6	51.2	1	65	100	81	Not significant
7	53.9	1	60	100	72	Not significant

**Table 5:** Quantification of bioactive compounds in peanut shell extracts.

	Content (mg/g-Biomass)
Total polyphenol	6.6 ± 0.05
Total flavonoid	4.1 ± 0.40
Luteolin	1.6 ± 0.02

**Table 6:** Evaluation of antioxidant and anti-elastase activity of peanut shell extracts.

	Luteolin Standard	Peanut Shell Extracts
FRAP value (mmol/L)	3.0 ± 0.08	4.3 ± 0.03
ABTS IC_50_ (µg/mL)	17.3 ± 0.08	4.6 ± 0.10
DPPH IC_50_ (µg/mL)	174.7 ± 0.34	11.0 ± 0.44
Anti-elastase (%)	49.9 ± 0.03	88.3 ± 0.55

**Table 7:** The carbohydrate composition of untreated and extracted peanut shells.

	Untreated Peanut Shells	Extracted Peanut Shells
Glucan (%)	23.2 ± 0.25	23.6 ± 0.10
Xylan (%)	9.8 ± 0.03	14.6 ± 0.03
Arabinan (%)	2.5 ± 0.06	1.0 ± 0.07
Others (%)	64.5	60.8

**Table 8:** Summary of luteolin extraction process from various biomass.

Biomass	Part	Extraction Method	Conditions				Luteolin Content (mg/g-Biomass)	Ref.
			Solvent	Temp. (°C)	Time (h)	S/L Ratio (g/L)		
Carrot	leaves	Hot water extraction	DW	120	0.2	15	0.8	[20]
Cretan brake fern	-	Ultrasound-assisted extraction	56.7% EtOH	74.3	0.8	33.7	0.7	[34]
Deulkkae	leaves	Supercritical fluid extraction	100% MeOH	25	1		0.1	[19]
Olive tree	leaves	Ultrasound-assisted extraction	100% MeOH	40	2	100	0.3	[35]
Olive tree	leaves	Pressurized liquid extraction	80% MeOH	190	0.1		2.7	[36]
Peanut	shells	Ultrasound-assisted extraction	100% MeOH	55	0.7	33.3	2.4	[18]
Pigeon pea	leaves	Enzyme-assisted extraction		30–35	18		0.3	[37]
Ribwort plantain	stem, fruit, leaves	Ultrasound-assisted extraction	45% EtOH	40	1.3	20	0.9	[38]
Peanut	shells	Maceration	70.0–97.5% MeOH	33.8–48.5	1	100	1.6	In this study

**Table 9:** Coded variables and their levels for the central composite design (CCD).

Variables	Unit	Symbol	Coded Factor Levels				
			−2	−1	0	1	2
Temperature	*X*_1_	°C	0	15	30	45	60
Time	*X*_2_	h	1	2	3	4	5
MeOH concentration	*X*_3_	%	0	25	50	75	100

## Data Availability

The data presented in this study are available on request from the corresponding author.
